# Biochemical characterization, structure-guided mutagenesis, and application of a recombinant D-allulose 3-epimerase from *Christensenellaceae bacterium* for the biocatalytic production of D-allulose

**DOI:** 10.3389/fbioe.2024.1365814

**Published:** 2024-02-27

**Authors:** Lijun Guan, Ling Zhu, Kunlun Wang, Yang Gao, Jialei Li, Song Yan, Xindi Zhang, Nina Ji, Jing Fan, Ye Zhou, Xinmiao Yao, Bo Li

**Affiliations:** ^1^ Heilongjiang Academy of Sciences, Institute of Food Processing, Harbin, China; ^2^ Key Laboratory of Food Processing of Heilongjiang Province, Harbin, China; ^3^ Heilongjiang Academy of Agricultural Sciences, Soybean Institute, Harbin, China

**Keywords:** D-allulose, D-allulose 3-epimerase, bioconversion, site-directed iteration mutagenesis, apple juice

## Abstract

D-Allulose has become a promising alternative sweetener due to its unique properties of low caloric content, moderate sweetness, and physiological effects. D-Allulose 3-epimerase (DAEase) is a promising enzyme for D-Allulose production. However, the low catalytic efficiency limited its large-scale industrial applications. To obtain a more effective biocatalyst, a putative DAEase from *Christensenellaceae bacterium* (CbDAE) was identified and characterized. The recombinant CbDAE exhibited optimum activity at pH 7.5°C and 55°C, retaining more than 60% relative activity from 40°C to 70°C, and the catalytic activity could be significantly increased by Co^2+^ supplementation. These enzymatic properties of purified CbDAE were compared with other DAEases. CbDAE was also found to possess desirable thermal stability at 55°C with a half-life of 12.4 h. CbDAE performed the highest relative activity towards D-allulose and strong affinity for D-fructose but relatively low catalytic efficiency towards D-fructose. Based on the structure-guided design, the best double-mutation variant G36N/W112E was obtained which reached up to 4.21-fold enhancement of catalytic activity compared with wild-type (WT) CbDAE. The catalytic production of G36N/W112E with 500 g/L D-fructose was at a medium to a higher level among the DAEases in 3.5 h, reducing 40% catalytic reaction time compared to the WT CbDAE. In addition, the G36N/W112E variant was also applied in honey and apple juice for D-allulose conversion. Our research offers an extra biocatalyst for D-allulose production, and the comprehensive report of this enzyme makes it potentially interesting for industrial applications and will aid the development of industrial biocatalysts for D-allulose.

## 1 Introduction

D-allulose, a C3 epimer of D-fructose, is a rare ketohexose found in some plants in small amounts. In spite of having 70% sweetness compared to sucrose (Bilal et al., 2018; Van Laar et al., 2021; Zhang et al., 2023), its caloric value is much lower because of its low bioavailability, making it an attractive, generally regarded as safe (GRAS) sugar substitute (Ran et al., 2019). Recent studies have also demonstrated that it possesses several additional health benefits, such as antioxidative, lipid-normalizing, hypoglycemic, and anti-obesity effects (Bober and Nair, 2019; Li et al., 2021). As a consequence, there is a considerable demand in the food industry for the cost-effective manufacturing of rare sugars such as allulose, as evidenced by the 2022 allulose market size estimate of USD 223.1 million. Because of the global diabetes issue and the growing importance of prebiotics, this industry is predicted to grow (Guo et al., 2021).

D-allulose can be chemically synthesized, but this route requires sophisticated purification processes and generates numerous chemical waste by-products (Hu et al., 2021), making it not economically viable. Many attempts have been made in recent years to synthesize D-allulose via a bioprocess based on the Izumoring strategy using enzymes from the ketose 3-epimerases (KEase) family (Wang et al., 2020; Patel et al., 2021; Hu et al., 2022). The D-allulose 3-epimerases (DAEases), which are responsible for the interconversion between D-fructose and D-allulose, were thought to be a promising biocatalyst in the KEase family (Li et al., 2019). Recently, an increasing number of DAEases have been cloned from various microorganisms, such as *Arthrobacter globiformis* (Yoshihara et al., 2017), *Clostridium cellulolyticum* (Mu et al., 2011a), *Arthrobacter psychrolactophilus* (Laksmi et al., 2022), *Agrobacterium tumefaciens* (Kim et al., 2006), *Pseudomonas* sp. ST-24 (Itoh et al., 1994), *Sinorhizobium fredii* (Li et al., 2023), *Staphylococcus aureus* (Zhu et al., 2019a), *Sinorhizobium* sp. (Zhu et al., 2019b), *Rhodobacter sphaeroides* (Qi et al., 2017) and so on. However, the intrinsic limitations of DAEases, such as their low catalytic efficiency and poor thermostability, hindered their further application in the food industry.

In recent years, protein engineering has emerged as a robust strategy to enhance the performance of biocatalysts by creating novel improved enzyme variants (Li et al., 2021b; Zhu et al., 2021; Li et al., 2022). Rational design and functional modification based on structure-function relationships have enabled a significant improvement of enzymatic performance in many recent studies. Li et al. (2023) achieved a 17-fold improvement in the catalytic efficiency of *S. fredii* DAEase using structure-guided rational design and directed evolution. Bosshart et al. (2013) revealed that a semi-rational surface engineering strategy for multimeric enzymes, based merely on a crystal structure and minimal screening work, could significantly improve their thermostability. Therefore, these approaches have been shown to be an effective strategy for modifying enzymes, allowing them to be tailored with various desired properties for industrial applications.

In this study, a putative D-allulose 3-epimerase from *Christensenellaceae bacterium* (CbDAE) was first identified and characterized. We predicted the three-dimensional (3D) structure of CbDAEase through homology modeling in SWISS MODEL. Then, in order to enhance the enzymatic performance, site-directed mutagenesis was carried out using homologous structures and sequence analyses of ketose 3-epimerase family enzymes. Furthermore, the biosynthesis of D-allulose from D-fructose using the best variant was also validated on a preparative scale, confirming its industrial application potential. Finally, in order to efficiently convert high-calorie D-fructose from food into D-allulose, apple juice, and honey were also successfully treated with CbDAE.

## 2 Materials and methods

### 2.1 Cloning, expression and purification of CbDAE

The coding sequence of a putative D-allulose 3-epimerase from *Christensenellaceae bacterium* (CbDAE) was codon-optimized and synthesized by AZENTA (Suzhou, China), after which it was ligated into the pET-28a (+) vector between the *Nde*I and *Eco*RI sites (Guan et al., 2021). The recombinant plasmid was introduced into *Escherichia coli* strain BL21 (DE3) for gene expression. The recombinant strain was grown at 37°C in lysogeny broth (LB) containing 50 mg/L kanamycin until the optical density (OD_600_) reached 0.6–0.8 (Gao et al., 2023). Then, isopropyl-β-D-thiogalactopyranoside (IPTG) was added to the culture at a final concentration of 0.1 mM to induce the overexpression of CbDAE at 16°C overnight. The Cells were harvested by centrifugation at 5,000 ×g and 4°C for 15 min, and then washed twice with 0.85% NaCl solution (Xu et al., 2016; Yoshihara et al., 2017). For the purification of CbDAE, the cells were disrupted in lysis buffer (20 mM Tris-HCl, 20 mM imidazole, and 500 mM NaCl, pH 8.0) for 10 min. Cell debris was removed by centrifugation at 40,000 ×g for 30 min at 4°C, and the supernatant was trapped on Ni-NTA Superflow resin (Qiagen, Hilden, Germany). The resin was washed with wash buffer (20 mM Tris-HCl, 50 mM imidazole, 0.5 M NaCl, and 1 mM DTT, pH 8.0) and then CbDAE was eluted with elution buffer (20 mM Tris-HCl, 500 mM imidazole, 500 mM NaCl and 1 mM DTT, pH 8.0). The purified protein was stored at 4°C for further experiments (Wu et al., 2019).

### 2.2 Enzyme activity assay of CbDAE

The protein concentration was measured using a BCA protein assay kit (Solarbio, Beijing) with bovine serum albumin as the standard. The specific activity of CbDAE was measured in a mixture containing 10 g L^−1^ D-fructose, 1 mM Co^2+^, and 0.06 mg/mL enzyme, at 60°C for 10 min. The reactions were stopped by boiling for 10 min and centrifuged to remove the denatured protein, after which the produced D-allulose was measured using high-performance liquid chromatography (HPLC, Agilent Technologies, Waldbronn, Germany) using a refractive index detector and a carbohydrate ES 5u column (5 μm, 4.6 × 250 mm, Agela Technologies, China). The mobile phase consisted of 80.0% acetonitrile and 20.0% water (v v^−1^) at a flow rate of 0.8 mL min^−1^.

### 2.3 The effects of temperature, pH, and metal ions on enzyme activity and stability

To assay the temperature profile of CbDAE, the reaction was carried out at 30°C–90°C. Moreover, the thermostability of CbDAE was monitored by incubating the purified enzyme in 50 mM Tris-HCl (pH 8.0) at 50, 55, 60, 65, and 70°C, followed by measurement of the residual activity. For determining the half-life time (t_1/2_) of CbDAE, the t_1/2_ values were calculated based on the first-order inactivation kinetic model. The purified enzyme was pre-incubated at 55°C. After extracting the incubated samples at given time intervals, the residual activities were determined under standard reaction conditions. The initial activities of the enzymes that were not incubated were set as 100%.

The optimal pH was determined by measuring the enzyme activity in 20 mM buffers, including MES-HCl (pH 5.5–6.5), K-phosphate (pH 7.0–8.0), Tris-HCl (pH 8.5–9.0), and glycine-NaOH (pH 9.0–10.0) at 60°C for 10 min. In order to determine the pH stability, the purified CbDAE was pre-incubated in the described buffers at 4°C for 4 h. The residual activity was then assayed in Tris-HCl buffer (pH 8.0) using 10 g L^−1^ D-allulose as the substrate at 60°C.

To investigate the effect of different metal ions, the enzyme activity assays were performed after treatment with EDTA overnight, followed by the addition of 1 mM Ba^2+^, Co^2+^, Cu^2+^, Mg^2+^, Ca^2+^, Zn^2+^, Mn^2+^, Ni^2+^, and Fe^2+^, respectively. When relative activities were analyzed, the enzyme activity of CbDAE at 60°C in Tris-HCl buffer pH 8.0 was defined as 100%.

All measurements were performed in three independent reactions, and the error bars indicated the standard deviations (*n* = 3).

### 2.4 Substrate specificity and kinetic properties of CbDAE

The substrate specificity of CbDAE was determined under standard catalytic reaction using 200 mM concentrations of different substrates—D-allulose, D-fructose, D-tagatose, and D-sorbose. The optimum enzyme activity was defined as 100%.

The kinetic parameters of CbDAE were determined by measuring the activity using different substrates with a concentration range of 5–300 mM, including D-fructose, D-allulose, D-tagatose, and D-sorbose. Kinetic parameters were calculated by following the Michaelis-Menten constant and Lineweaver-Burk plot analysis protocols.

### 2.5 Structural modeling of CbDAE

The three-dimensional (3D) homology model of CbDAE was predicted using SWISS MODEL (https://swissmodel.expasy.org/) using the crystal structure of CcDAE from *Clostridium cellulolyticum* (PDB ID: 3VNI) as the template (Chan et al., 2012; Wang et al., 2020). The best model was judged by the values of the DOPE assessment scores and the Modeler objective function. To obtain a model of the enzyme-substrate complex, molecular docking was performed using AutoDock software (Eberhardt et al., 2021). The 3D structure of the substrate D-fructose was downloaded from the PubChem website (http://pubchem.ncbi.nlm.nih.gov). Hydrogens atoms were added to obtain the protonated 3D model which was further used as the receptor. PyMOL (http://www.pymol.org) was used to visualize and analyze the generated model structure (Seeliger and de Groot, 2010).

### 2.6 Mutagenesis of CbDAE

Site-directed mutagenesis was performed via polymerase chain reaction (PCR) using the KOD plus mutagenesis kit (Toyobo, Tokyo, Japan) with the pET22b-CbDAE plasmid as the template. The primers were designed using QuickChange Primer Design (Agilent, United States) and are listed in Supplementary Table S1. After the PCR reaction was complete, the template was removed by digestion with *Dpn*I, and the product was further cyclized by T4 polynucleotide kinase and Ligation High DNA ligase. The mutations were confirmed by DNA sequencing (AZENTA, Suzhou, China). The plasmids harboring the correct mutated sequences were introduced into *E. coli* BL21 (DE3) for expression of the mutant proteins.

### 2.7 Bioconversion of D-fructose into D-allulose

The synthesis of D-allulose was conducted in 5 mL reaction systems containing 0.06 mg/mL purified CbDAE with 500 g L^−1^ of D-fructose under the optimal conditions, and samples were taken at regular time intervals. Apple juice and honey were procured from the local market. The honey sample was diluted with 4 times of distilled water. The pH of the fruit juice and diluted honey sample were adjusted to 7.5 using 1 M NaOH before use. The concentrations of D-allulose and D-fructose were quantified by HPLC.

## 3 Results and discussion

### 3.1 Sequence analysis and structure modeling of CbDAE

**FIGURE 1:**
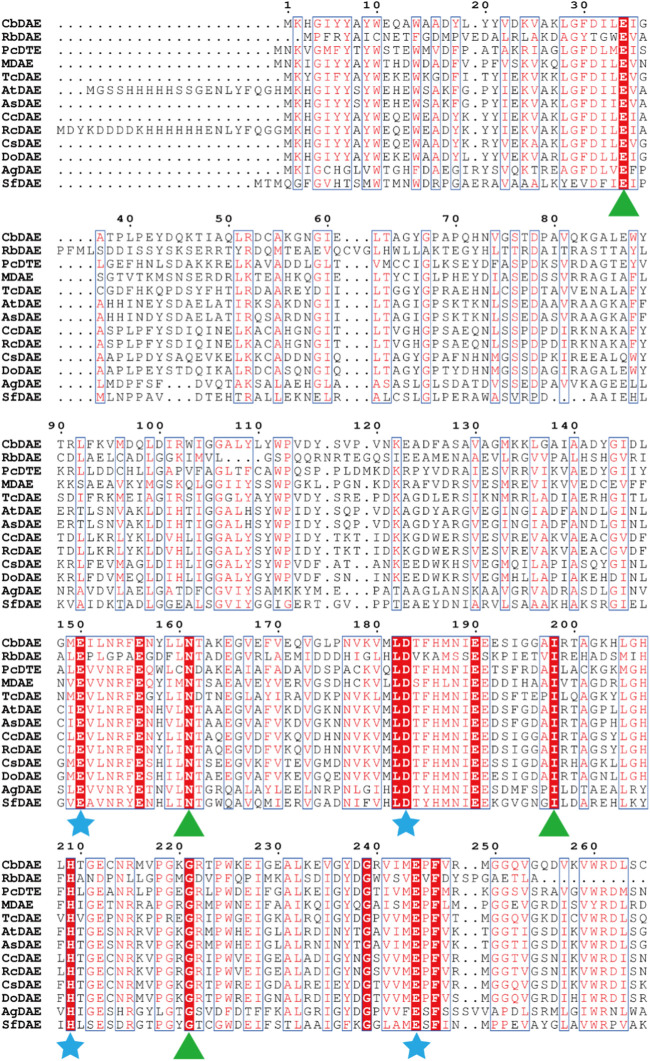
Multiple sequence alignment of CbDAE from different bacteria. The star and triangle indicate the extremely conserved residues involved in the metal coordinating sites and involved in the binding sites with substrate, respectively.

**FIGURE 2:**
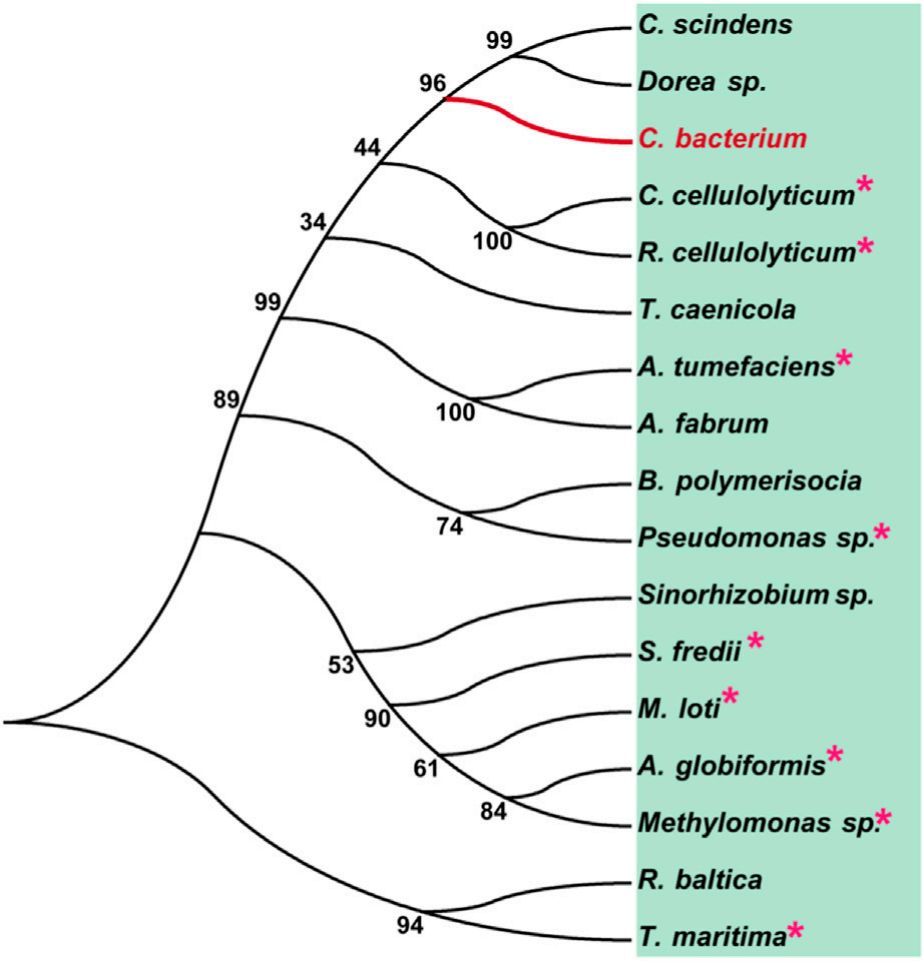
Phylogenetic tree of DAEase from *Christensenellaceae bacterium* (CbDAE) and homologous enzymes. DAEases with similar amino acid sequences to CbDAE were searched in the UniProt and NCBI databases, and the phylogenetic tree was constructed using MEGA 7.0. Enzymes with solved crystal structures are indicated with pink stars.

To analyze the overall sequence and conserved amino acids of CbDAE, multiple sequence alignment was conducted based on the protein sequences of characterized DAEase family enzymes from the other organisms. As shown in Figure 1, CbDAE showed the highest sequence similarity with the DAEase from *C. cellulolyticum* (62.2%) (Chan et al., 2012), *A. tumefaciens* (58.3%) (Kim et al., 2006), and *T. caenicola* (52.8%) (Nagano et al., 2002). Additionally, CbDAE shared 36.8% sequence similarity with the DTEase from *Pseudomonas* sp. (Itoh et al., 1994), but only 25.4% and 24.2% sequence similarity with the LREases from *Methylomonas* sp. (MdLRE) (Yoshida et al., 2021) and *T. maritima* (TmLRE) (Shin et al., 2017), respectively. Notably, the residues E150, D183, H209, and E244, which are involved in substrate recognition and metal coordination, were absolutely conserved with those of other reported DAEases. In addition, substrate O-1, O-2, and O-3 binding sites (E34, N161, I198, and G221) of CbDAE were also completely conserved. The conserved amino acid residues indicated that the catalytic mechanism of CbDAE was similar to that of other DAEase family members. Furthermore, a phylogenetic tree of KEase family enzymes was constructed using MEGA 7.0, which also indicated that CbDAE belongs to the DAEase family, and may have catalytic activity (Figure 2).

**FIGURE 3:**
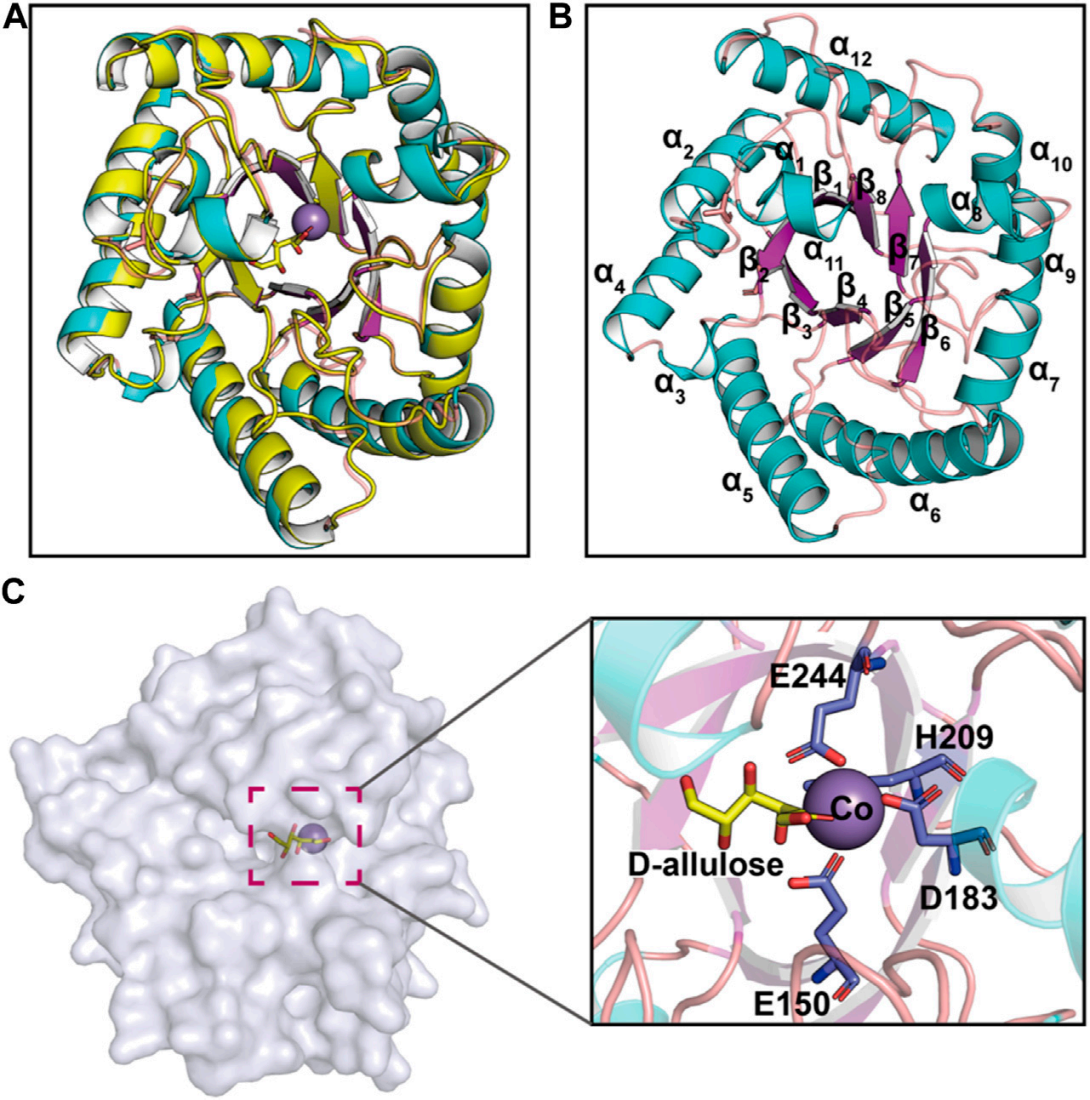
Cartoon representation of the CbDAE homology model. **(A)** The 3D-structure of CbDAE was generated through homology modeling in SWISS MODEL, based on the crystal structure of CcDAE from *C. cellulolyticum* (PDB code: 3VNI). **(B)** The overall structure of CbDAE. The core β-strands of the TIM barrel domain are shown in magenta, while the surrounding helices and loops are shown in cyan and wheat, respectively. **(C)** Surface representation of the overall structure and enlarged view of the catalytically active pocket of CbDAE. The catalytic residues are shown as slate sticks. Co^2+^ is shown as a light-blue sphere, and the substrate D-allulose is shown as yellow sticks.

Recent advances in structure prediction have simplified the structural modeling of enzymes. A 3D homology model of CbDAE was generated based on the crystal structure of DAEase from *C. cellulolyticum* (PDB code 3VNI) (Figure 3A). The structure of CbDAE was composed of eight β-strands, twelve α-helices, and multiple loop regions (Figure 3B). The CbDAE monomer possessed a typical TIM-barrel (β/α)_8_ domain at the conserved core structure. To obtain the model of the enzyme-substrate complex, molecular docking was performed in AutoDock software. The homology model of CbDAE indicated that the four residues Glu150, Asp183, His209, and Glu244 formed a catalytic tetrad and coordinated the Co^2+^ ion in the active pocket (Figure 3C). The catalytic residues were analogous to the template crystal structure of CcDAE, indicating that the reaction mode of this enzyme was similar to that of other family members.

### 3.2 Biochemical characterization of CbDAE

**FIGURE 4:**
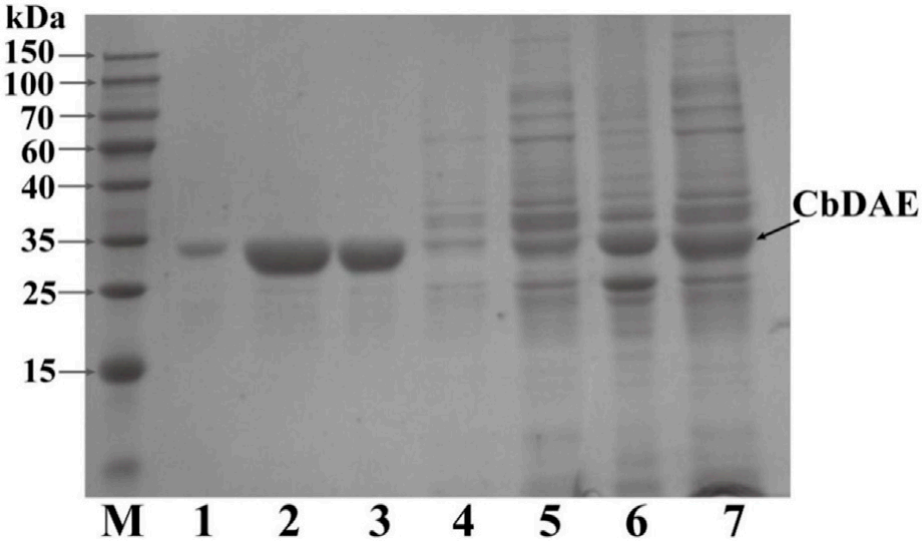
The SDS-PAGE analysis of the expression and purification of recombinant CbDAE. *Lane M*: protein molecular weight markers; *Lanes 6-7*: crude extract; *Lane 5*: flowthrough; *Lane 4*: unbound proteins; *Lane 3*: the sample before elution; *Lane1-2*: fractions eluted from His affinity resin. Gels with 12% acrylamide gels used to resolve the proteins in the mixtures.

**FIGURE 5:**
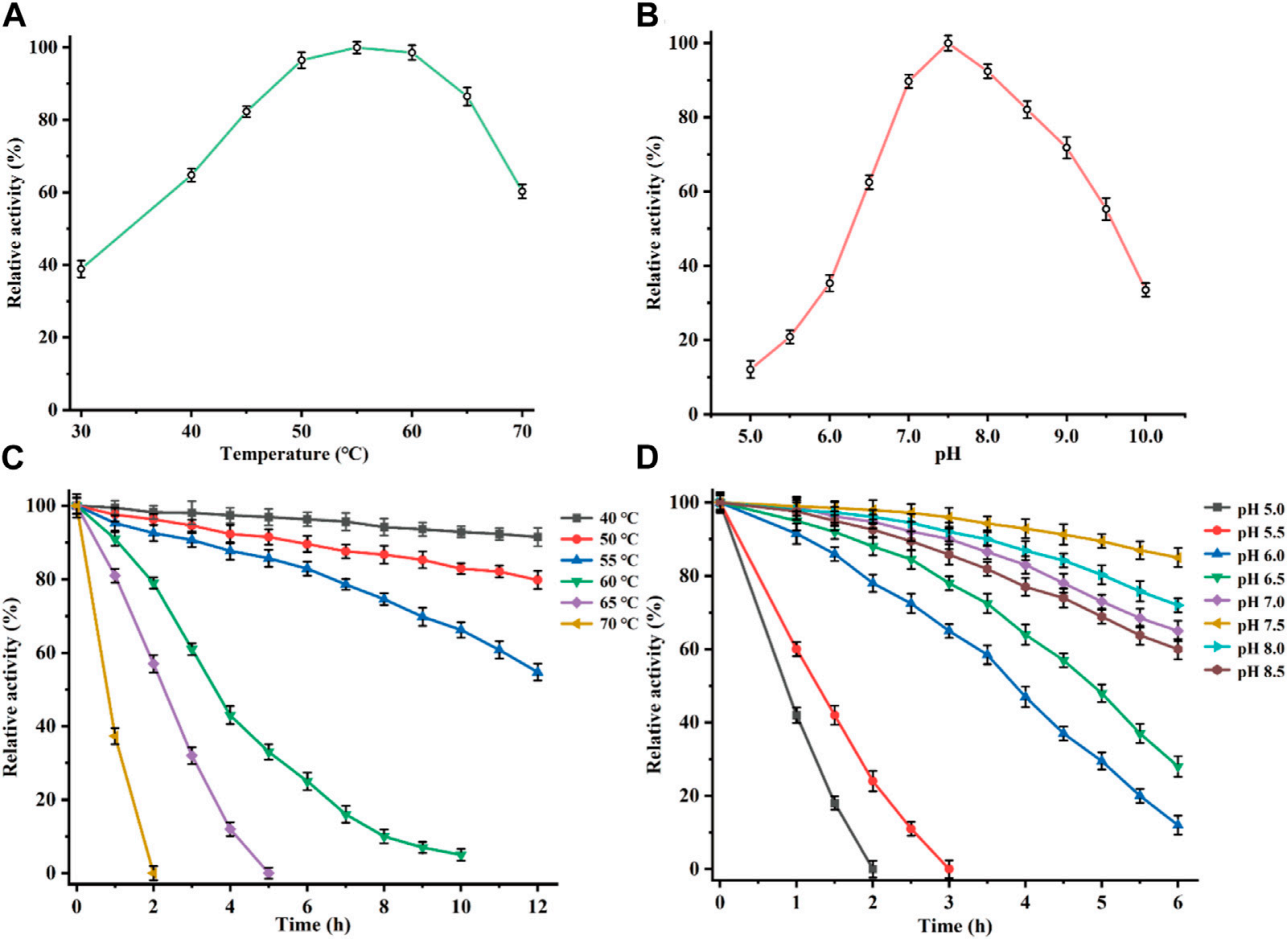
Determination of the optimal reaction conditions. Effects of temperature **(A)**, and pH **(B)**, on the relative activity of CbDAE. **(C)** The thermostability of CbDAE. **(D)** The pH stability of CbDAE. All activity assays were performed in triplicate, and the values represent the means ± standard deviations.

The recombinant CbDAE was successfully overexpressed in *E. coli* BL21 (DE3) (Figure 4). To evaluate the catalytic performance of CbDAE, the temperature and pH profiles were recorded. As shown in Figure 5A, the optimal temperature for the epimerization activity of CbDAE was 55°C, and it maintained over 80% relative activity in the temperature range of 45°C–65°C. Thermal stability is a significant index for industrial enzymes and is related to their commercial applications. Thermal stability was therefore investigated by measuring the relative activities of CbDAE after incubation at temperatures ranging from 40°C to 70°C (Figure 5B). The results indicated that the recombinant CbDAE was stable at temperatures of 40°C–55°C, retaining 80% of the initial activity after incubation for 5 h. However, the enzyme was completely inactivated after incubation at 65°C for 4.5 h. Based on these findings, the optimal reaction temperature for CbDAE was determined to be 55°C. The half-life (t_1/2_) of CbDAE at 55°C was 12.4 h, higher than the 9.5 h and 2.2 h of DAE from *Clostridium cellulolyticum* H10 (Mu et al., 2013) and DAE from *Thermoclostridium caenicola* (Chen et al., 2021), respectively. The t_1/2_ of CbDAE at 60°C was 3.9 h, higher than the 15 min of DAE from *Clostridium* sp. BNL1100 (Mu et al., 2011b) but lower than the 6.8 h of DAE from *Clostridium cellulolyticum* H10 (Mu et al., 2013). It is indicated that the CbDAE possessed relative desired thermal stability.

Subsequently, the effect of pH on CbDAE was investigated by assaying its activity over a wide pH profile from 5.0 to 10.0. The results showed that the optimal pH of CbDAE was 7.5, and it retained over 50% of residual activity in a pH range from 6.5 to 9.5 (Figure 5C). Moreover, the pH stability of CbDAE was measured after incubation at various pH values for 6 h. The results indicated that CbDAE was more stable at pH 7.0–8.5 and maintained more than 60% residual activity after incubation for 6 h (Figure 5D). However, the activity decreased dramatically at pH values below 5.5, and the enzyme was completely inactivated after 2 h at pH 5.0. These results are consistent with the previous reports, and the optimal reaction conditions of CbDAE were similar to those of most DAEase family enzymes.

**FIGURE 6:**
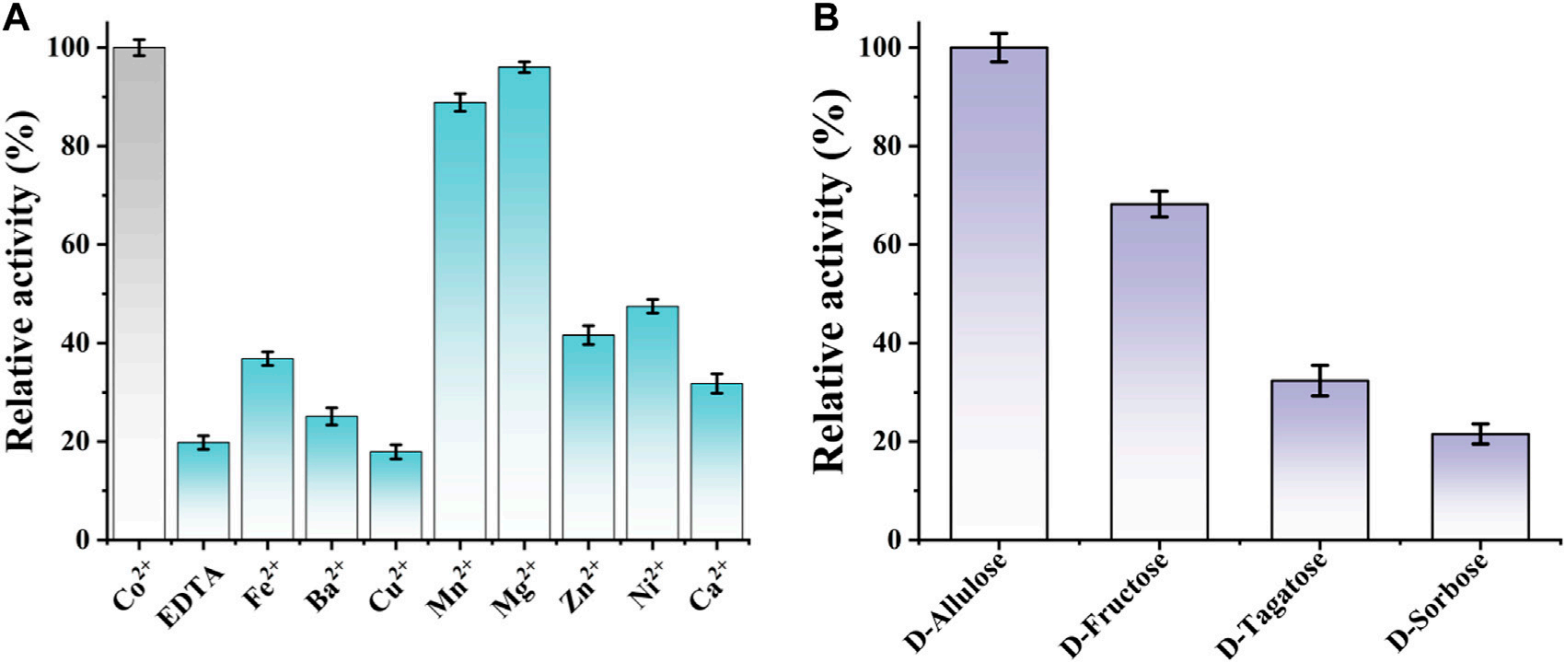
**(A)** The effect of various mental ions on the relative activity of CbDAE. **(B)** The substrate specificity of CbDAE.

Additionally, the effect of divalent metal ions on CbDAE activity was also determined using different mental ions at a final concentration of 1 mM after EDTA treatment. As shown in Figure 6A, it is indicated that CbDAE was a metal-dependent enzyme. The optimum metal ion for CbDAE was Co^2+^, and this maximum epimerization activity was defined as a relative activity of 100%. When Co^2+^ was replaced by Mg^2+^, Mn^2+^, Ni^2+^, and Fe^2+^, the relative enzyme activities were 96%, 86%, 46%, and 38%, respectively. In addition, CbDAE after EDTA treatment without metal ion supplementation displayed low (19%) catalytic activity, and Cu^2+^, Ba^2+^, and Ca^2+^ could not enhance the activity of the EDTA pre-incubated CbDAE. Some DAEases were strictly Co^2+^ or Mn^2+^ metal-dependent and showed not any activity without ions. Some, such as CbDAE, were not strictly metal-dependent, but the catalytic activity could be significantly increased by Co^2+^ or Mg^2+^ supplementation. These results were similar to previously characterized DTE or DAE family enzymes, which mostly exhibited higher activity in the presence of Co^2+^, Mg^2+^, and Mn^2+^, while Cu^2+^, Ca^2+^, and Zn^2+^ almost invariably decreased the catalytic activity.

### 3.3 Substrate specificity and kinetic properties of CbDAE

The substrate specificity of CbDAE was measured under the standard reaction conditions using D-allulose, D-fructose, D-tagatose, or D-sorbose as the substrate. The highest catalytic performance of CbDAE was observed with D-allulose, but it also showed moderate relative activity toward D-fructose (68.2%) and D-tagatose (32.6%), respectively (Figure 6B). By contrast, CbDAE had the lowest catalytic performance toward D-sorbose and only showed 20.9% relative activity compared to D-allulose. Given that D-allulose was a better substrate than D-tagatose, CbDAE could be categorized as a D-allulose 3-epimerase (DAEase). The result above was similar to previously reported DAE family enzymes that specifically favor D-allulose, such as the DAEs from *C. cellulolyticum* (Mu et al., 2011a), *Rhodopirellula baltica* (Mao et al., 2020) and *S. aureus* (Zhu et al., 2019a), which shared the highest substrate preference against D-alllulose and relatively lower activity against other sugars. In addition, it can be seen that CbDAE displayed higher relative epimerization activity on D-frutcose (68.2%) than that of Bp-DAE (33.4%) from *Blautia produca* (Tang et al., 2022), Ap-DAE (40%) from *A. psychrolactophilus* (Laksmi et al., 2022), *T. caenicola* DAEase (48.7%) from *T. caenicola* (Chen et al., 2021), but lower than that of DaeM from *Thermal aquatic habitat* (73.5%) (Patel et al., 2020).

The kinetic parameters of CbDAE with the abovementioned substrates were also measured under the standard reaction conditions, and the corresponding values were calculated via non-linear fitting to the Michaelis-Menten equation (Table 1). The *K*_m_, *k*_cat_, and *k*_cat_/*K*_m_ values of CbDAE for D-allulose were 72.8 s^−1^, 44.9 mM, and 1.62 s^−1^ mM^−1^, respectively, which had the highest catalytic activity compared to other substrates.

**TABLE 1:** The kinetic parameters and specific activities of CbDAE.

Substrate	*k*_cat_ (s^−1^)	*K*_m_ (mM)	*k*_cat_/*K*_m_ (s^−1^ mM^−1^)
D-Allullose	72.8 ± 6.5	44.9 ± 5.1	1.62 ± 0.24
D-Fructose	51.2 ± 3.8	46.5 ± 3.4	1.10 ± 0.09
D-Tagatose	27.4 ± 3.6	52.7 ± 6.2	0.52 ± 0.06
D-Sorbose	10.3 ± 1.5	61.6 ± 4.8	0.17 ± 0.03

^1^Data represent the mean ± standard deviation from three independent experiments.

The *K*_m_ value of CbDAE for D-fructose was 46.5 mM, lower than 94.5 mM of T. caenicola DAEase from *T. caenicola* (Chen et al., 2021), 141.3 mM of DaeM from *Thermal aquatic habitat* (Patel et al., 2020) and 237.5 mM of Bp-DAE from *B. produca* (Tang et al., 2022), showing stronger affinity for D-fructose. By contrast, the *k*_cat_/*K*_m_ value of CbDAE for D-fructose was 1.10 s^−1^ mM^−1^, which was lower than the 2.2 s^−1^ mM^−1^ of DAE from *T. caenicola* (Chen et al., 2021) and 3.3 mM^−1^ of DAE from *Dorea* sp. CAG317 (Zhang et al., 2015). It indicated that CbDAE was a potential biocatalyst for the synthesis D-allulose from D-fructose. Nevertheless, the relatively poor catalytic efficiency of CbDAE would be an obstacle to its further industrial application.

### 3.4 Construction of semi-rational CbDAE mutants

**FIGURE 7:**
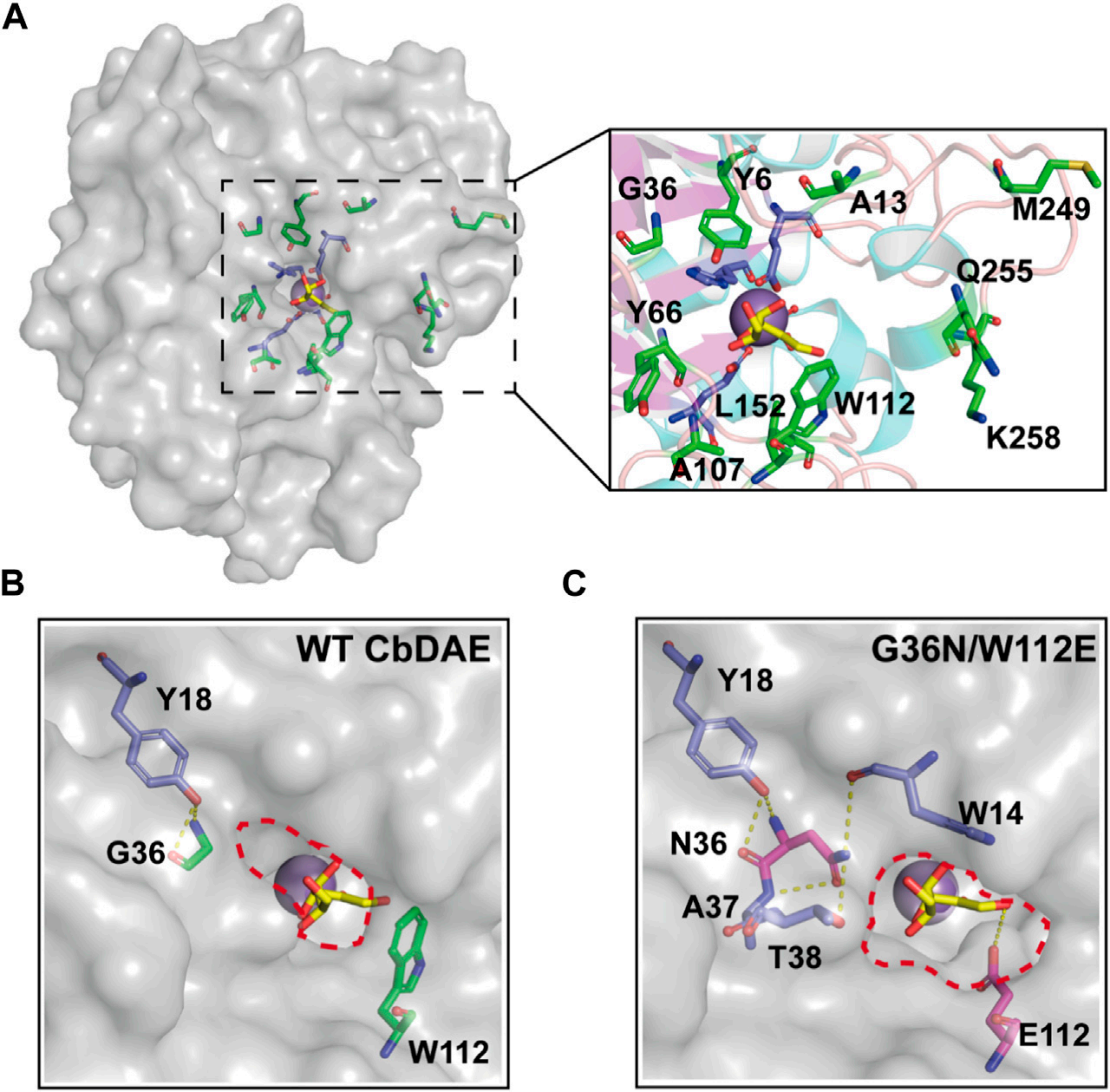
Structural representation at the substrate access tunnel of CbDAE. **(A)** Structural analysis of the active-site pocket of WT CbDAE. Comparison of the active-site pockets of WT CbDAE **(B)** and the best variant G36N/W112E **(C)**.

Directed evolution and rational design by site-directed mutagenesis are powerful strategies for creating enzymes with enhanced properties. According to the structural analysis and molecular docking, ten residues (Y6, A13, G36, Y66, A107, W112, L152, M249, Q255, and K258) that were situated within a distance of 5 Å from the substrate-binding site were chosen for rational design to further enhance the catalytic activity of CbDAE (Figure 7A). Saturation mutagenesis was then performed on these residues based on degenerate NNK codons. The high-throughput screening method based on a continuous spectrophotometric assay (CSA) in 96-well plates was employed to obtain the advantageous mutants (Li et al., 2021b). Then, in order to appropriately assess the catalytic performance of improved variants, the kinetic parameters were determined. The screening results revealed that the *k*_cat_/*K*_m_ value of mutant G36N was 1.94 s^−1^ mM^−1^, which was a 76% increase in catalytic efficiency compared with the WT CbDAE. Notably, the W112E mutant exhibited 3.94-fold higher activity than WT CbDAE (4.34 vs. 1.10 s^−1^ mM^−1^). After the initial round of screening, the mutant G36N was employed as the template for site-saturated mutagenesis at the W112 site. As expected, the double mutant G36N/W112E resulted in a most remarkable improvement of catalytic activity. The purified superior variant (G36N/W112E) showed a higher activity toward D-fructose with an affinity of 24.7 mM and catalytic efficiency of 4.63 s^−1^ mM^−1^ compared to the WT CbDAE. In addition, no obvious change of thermal stability was observed between the double mutant and WT CbDAE (Table 2).

**TABLE 2:** Comparison of kinetic parameters and thermal stability.

CbDAE	*K*_cat_ (s^−1^)	*K*_m_ (mM)	*k*_cat_/*K*_m_ (s^−1^ mM^−1^)	t_1/2_ (h)
WT	51.2 ± 3.8	46.5 ± 3.4	1.10 ± 0.09	12.4
G36N/W112E	116.2 ± 12.1	24.7 ± 0.3	4.63 ± 0.53	12.6

To further explore the molecular mechanisms underlying the enhancement of catalytic performance, the structural changes of the double mutant were examined. According to the structural analysis, Gly36 was situated close to the active pocket of the enzyme and at the substrate channel, where it formed two hydrogen bonds with other residues of WT CbDAE. By contrast, five hydrogen bonds were observed in Asn36 (Figure 7B). These differences suggested that the improved catalytic efficiency of G36N was likely due to the greater affinity of the protein for the substrate via the expanded hydrogen bond interaction network, which was consistent with the shift of *K*_m_ value (Table 2). Additionally, bulky Trp112 was changed to the smaller residue Glu, increasing the substrate-accommodating capacity of the active-site pocket. This finding indicated that the catalytic pocket of the mutant was easier for the substrate to access than that of WT (Figure 7C), which probably seemed to enhance the *k*cat value of the enzyme (Table 2).

### 3.5 Bioconversion of D-allulose using WT CbDAE and the best variant

**FIGURE 8:**
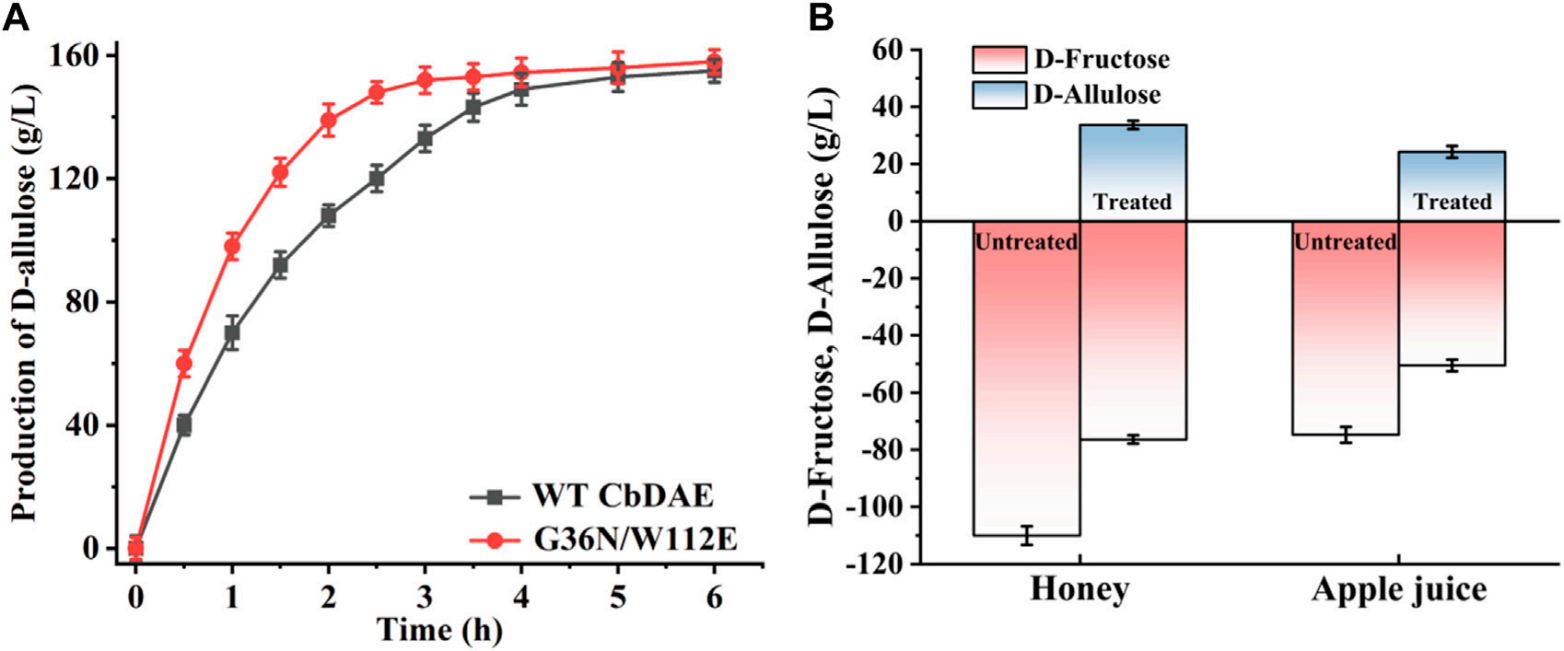
**(A)** Synthesis of D-allulose using the CbDAE enzyme with 500 g/L D-fructose as the substrate. **(B)** Bioconversion of D-fructose in apple juice and honey into D-allulose using the G36N/W112E variant.

To test the practical applicability of the WT CbDAE and its enhanced variant, both of them were used to biosynthesize D-allulose from D-fructose. The epimerase reaction is a reversible reaction, and the conversion yield of D-allulose was typically between 20% and 33% by employing different DAEases. When using 500 g/L D-fructose as the substrate, D-allulose titers of 151 g/L (conversion of 30.2%) and 155 g/L (conversion of 31%) were produced by the WT enzyme and the mutant G36N/W112E after reaching equilibrium, respectively. These results indicated that the catalytic performance of CbDAE with 500 g/L D-fructose was at a medium to a higher level. Significantly, the G36N/W112E variant displayed a faster catalytic rate, probably due to the high binding affinity of the D-fructose and catalytic efficiency, reaching the steady state after 3.5 h, while the WT required 5.5 h (Figure 8A). Li et al. (2021a) reported that isolated DAE enzyme from *Pirellula* sp. SH-Sr6A generated 152.7 g/L D-allulose using 500 g/L D-fructose as the substrate after 3 h (conversion rate of 30.5%). Zhang et al. (2018) reported that the bioconversion of 500 g/L D-fructose by DAEase from *Dorea* sp. CAG317 yielded approximately 120 g/L D-allulose after 3 h, corresponding to a conversion rate of 24%. Although there was only a slight difference in production between WT CbDAE and the double mutant when they were in phase equilibrium, the high catalytic rate of the double-variant could reduce 40% catalytic reaction time, which is very meaningful for industrial production. These results indicated that the G36N/W112E mutant has great potential for industrial production of D-allulose.

**FIGURE 9:**
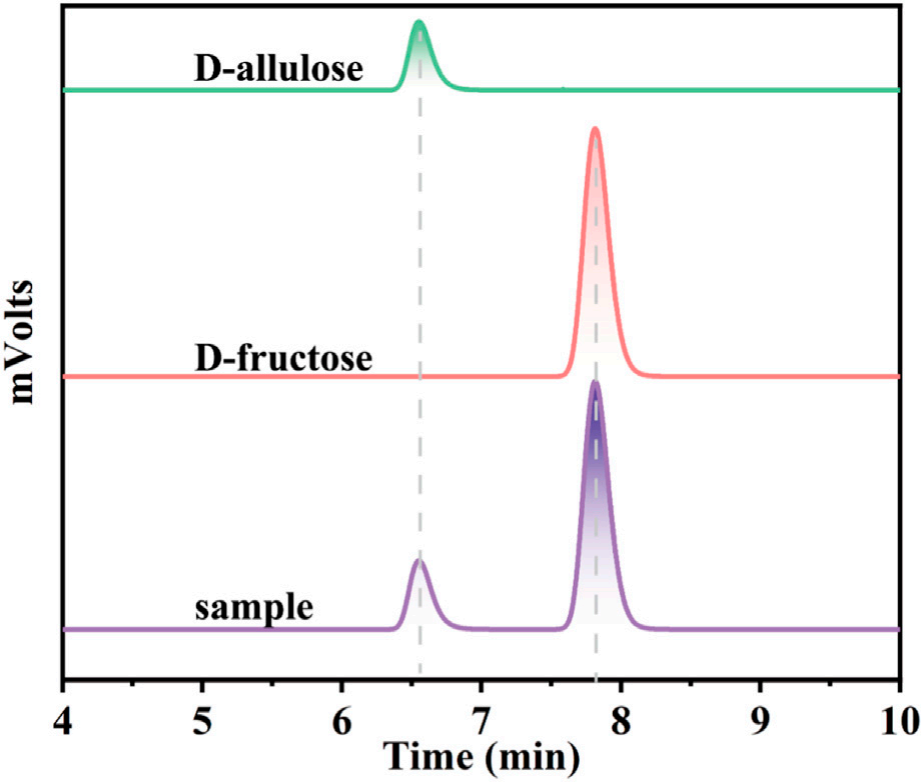
HPLC analysis of the products of CbDAE.

To further expand the practical application scenarios of the best variant, apple juice, and honey were selected as representative substrates due to their high content of D-fructose. Under the optimal reaction conditions, the G36N/W112 variant was able to effectively convert the D-fructose in apple juice and honey into D-allulose. Based on HPLC determination, the D-fructose content of honey and apple juice was 110.3 and 81.2 g/L, respectively (Figure 9). As shown in Figure 8B, 32.3 and 23.8 g/L of D-allulose were produced using the G36N/W112E variant, and the conversion rate was approximately 30.0%. The results illustrate the comprehensive application potential of the G36N/W112E variant for converting D-fructose in food into D-allulose and further demonstrate the feasibility of generating functional foods for specific populations.

## 4 Conclusion

In brief, a D-allulose 3-epimerase from *Christensenellaceae bacterium* (CbDAE) was identified and successfully cloned. Then, it was overexpressed in *E. coli* BL21 (DE3), purified and characterized. The optimum catalytic activity was observed at pH 7.5°C and 55°C, and the enzyme maintained more than 60% relative activity in the temperature range of 40°C–70°C. The recombinant CbDAE possessed desirable thermal stability at 55°C with a half-life of 12.4 h. In addition, it was not strictly metal-dependent, but the catalytic activity could be significantly increased by Co^2+^ supplementation. The substrate specificity and kinetic properties analysis showed that CbDAE performed the highest relative activity towards D-allulose and a strong affinity for D-fructose. Moreover, structure-guided site-saturation mutagenesis and a further round of combinatorial mutagenesis yielded the improved variant G36N/W112E with excellent catalytic performance. The G36N/W112E variant displayed a 4.21-fold enhancement of catalytic activity compared to the WT CbDAE using D-fructose as the substrate. The catalytic production of G36N/W112E with 500 g/L D-fructose was at a medium to a higher level among the DAEases, reducing 40% catalytic reaction time compared to the WT CbDAE. Finally, apple juice and honey were employed to assess the practical application of CbDAE and its superior variant. These research findings provided a valuable biocatalyst that will promote the industrial production of D-allulose.

## Data Availability

The original contributions presented in the study are included in the article/Supplementary material, further inquiries can be directed to the corresponding author.
