# Supplementary material for: Biochemical characterization, structure-guided mutagenesis, and application of a recombinant D-allulose 3-epimerase from Christensenellaceae bacterium for the biocatalytic production of D-allulose
Source: Front Bioeng Biotechnol. 2024 Feb 27;12:1365814. doi: 10.3389/fbioe.2024.1365814 (PMC10927987; doi:10.3389/fbioe.2024.1365814)
Supplement: Supplementary file 1 [file Table1.docx]

# Supplementary material

# Biochemical characterization, structure-guided mutagenesis, and application of a recombinant D-allulose 3-epimerase from *Christensenellaceae bacterium* for the biocatalytic production of D-allulose

**Table. S1** Primers used for the mutant construction in this article.

| Primer name | Sequence of primer |
| --- | --- |
| G36N-F | ATTAACGCGACCCCGCTGC |
| G36N-R | TTCCAGAATATCAAAGCCCAGTTTCG |
| W112E-F | TATGAGCCGGTGGATTATAGCGTGC |
| W112E-R | CAGATACAGCGCGCCGCCAATCCAGC |
